# Emerging shadow health systems: regulating health-focused generative AI chatbots from a European perspective

**DOI:** 10.3389/fpubh.2026.1830068

**Published:** 2026-06-24

**Authors:** Hannah van Kolfschooten, Tjaša Petročnik

**Affiliations:** 1 Centre for Life Sciences Law, Faculty of Law, University of Basel, Basel, Switzerland; 2 Tilburg Institute for Law, Technology, and Society, Tilburg University, Tilburg, Netherlands

**Keywords:** AI regulation, ChatGPT, ChatGPT Health, digital public health, generative AI, health chatbots, health equity, health law

## Abstract

ChatGPT Health and other health-focused generative AI chatbots increasingly function as alternative first points of contact that may mediate—and in some cases substitute—engagement with regulated healthcare systems. At scale, these systems can shape care-seeking behavior, system capacity, trust in clinical expertise, and health equity. We describe this development as the emergence of shadow health systems: privately governed infrastructures that perform health-system functions without being subject to healthcare-specific safeguards. EU law currently regulates such tools based on declared medical purpose rather than their real-world effects, creating gaps in legal protection. This Policy Brief examines the public health implications and regulatory positioning of health-focused generative AI chatbots in Europe and proposes targeted reforms to address emerging governance gaps.

## 1 Introduction

Consumer-facing artificial intelligence (AI) chatbots are rapidly becoming a primary interface through which individuals seek and act upon health information. OpenAI reports that more than 230 million people ask ChatGPT health-related questions each week (1), on topics such as diagnosis (2), medical treatment (3), medication (4), and wellbeing and lifestyle issues (5, 6). In January 2026, OpenAI launched ChatGPT Health in the United States (US), a health-focused addition to its generative AI chatbot designed to integrate users’ personal health data and generate personalized insights. Similar initiatives by competitors such as Anthropic, Microsoft, and Google’s Fitbit further indicate a growing market interest in generative AI chatbots for personal health-related use (7–9). The emergence of ChatGPT Health should therefore not be understood as an isolated product development. Rather, it points to a broader shift in which major providers of generative AI are increasingly building private health-related infrastructures.

This development in the first place reflects a shift in how people engage with health information. By integrating longitudinal personal data into continuous conversational interfaces, these AI chatbots can function as alternative first points of contact that may mediate – and in some cases substitute for – engagement with regular healthcare pathways. They may influence whether, when, and how individuals access professional services, yet operate outside the legal, ethical, and institutional frameworks that govern formal healthcare (10), exposing a broader infrastructural transformation in the background. In other words, these conversational AI systems effectively constitute “shadow health systems”: privately governed infrastructures that increasingly mediate – and sometimes substitute – engagement with regulated healthcare. Unlike telemedicine or digital health platforms that largely operate within regulated healthcare systems, these infrastructures function outside formal care pathways and are thus not embedded in corresponding legal and institutional safeguards (11). Although ChatGPT Health is not yet available in the European Union (EU), the United Kingdom (UK), and Switzerland, its likely roll-out in Europe raises pressing regulatory questions. Where scarcities in formal healthcare make such tools especially attractive, it becomes even more important to examine the risks of shadow health systems before they become normalized alternatives to regulated care.

Against this backdrop, this Policy Brief uses doctrinal and policy analysis to examine the public health implications of a potential European launch of ChatGPT Health and comparable health-focused generative AI chatbots through a regulatory lens. These tools may reduce barriers to health information and care, improve access to health information, provide low-threshold support, and offer early guidance, particularly for individuals facing barriers to traditional healthcare services. While thus fulfilling an important public health function, they also raise concrete and significant concerns regarding safety, data governance, privacy, bias, accountability, and equity (12). Importantly, these effects are structural: they extend beyond individual users and may shape population-level health outcomes (13, 14). We outline how the current EU legal approach creates regulatory gaps and propose measures to align legal obligations with potential real-world effects of health-focused generative AI chatbots. While this analysis focuses on the European regulatory context, similar developments are emerging elsewhere, making the analysis relevant also in a wider context.

## 2 From ChatGPT to ChatGPT Health: What is new?

ChatGPT Health illustrates a broader shift in the role of generative AI chatbots in population health. Unlike general-purpose chatbots, which users may consult for health questions, it is designed specifically for health-related interaction and can connect to medical records and health applications to generate responses grounded in longitudinal personal data. As Table 1 shows, both tools rely on the same underlying model. The novelty of ChatGPT Health therefore lies not in the model itself but in its integration with personal health data infrastructures and its repositioning as a personalized health interface.

**Table 1:** Functional differences between general-purpose ChatGPT and ChatGPT Health.

Feature	ChatGPT (standard)	ChatGPT Health
Purpose	General-purpose AI assistant across domains	Dedicated health & wellness environment within ChatGPT
Information sources	Pre-trained model knowledge + user input in chat	Same pre-trained model + user input + optional connected medical records & health apps
Personalization	Limited to what user types and saved memory (if enabled)	Grounded in structured, longitudinal personal health data (when connected)
Evaluation approach	General cross-domain safety and quality testing	Health-specific evaluation framework (physician-informed, clinically aligned criteria—“HealthBench”)
Privacy architecture	Standard ChatGPT privacy controls	Separate, isolated health space with enhanced encryption; health chats not used for model training
Regulatory positioning	General AI assistant, not a medical device	Still not a medical device, but optimized for health-related support

These differences change the system’s functional role. Integrating personal health data allows personalized health responses rather than providing general health information. Personalized outputs can shape how users interpret symptoms and make health-related decisions, while the system remains formally positioned as an informational tool. If individuals use these systems to seek health advice at scale, their significance extends beyond individual use to structural effects on public health and health system governance.

## 3 Public health implications of health-focused generative AI chatbots

By functioning as alternative first points of contact that may mediate – and in some cases substitute – engagement with regulated care, health-focused generative AI chatbots may reframe how individuals interpret symptoms, seek care, and whom they trust. This may lead to structural effects for public health.

First, when AI systems function as first points of contact with healthcare, they can reshape how and when patients access regulated services, with consequences for capacity and safety. If a chatbot frames mild symptoms as potentially serious, precautionary consultations may increase, adding pressure to already stretched primary and emergency care (15). Conversely, if serious symptoms are under-triaged, engagement with health services may be delayed, resulting in worse clinical outcomes or more costly interventions down the line (16). Both cases distort patient flows and misallocate healthcare resources (15), impacting healthcare systems’ capacities to deal with actual health needs.

Second, AI chatbots may begin to shape not only access patterns but also epistemic authority in healthcare. Because chatbot responses are personalized and seem confident, users may rely on them as if they were clinical advice, even when providers disclaim to be providing medical advice (17). At the same time, recent research shows that AI chatbots prioritize being helpful over being accurate in medical contexts, which might produce convincing but false medical information or validate users’ harmful health attitudes and behaviors (18). Emerging studies further indicate variability in triage accuracy and user reliance on chatbot-generated health advice, reinforcing concerns about their potential impact on care-seeking behavior (19–21). At scale, this can be detrimental for public health. Over time, this might also generate competing sources of authority: regulated clinical expertise on the one hand and privately governed AI advice on the other. Diverging advice might strain patient-doctor relationships, both due to accuracy disputes and due to AI actively shaping care expectations (22).

Third, widespread use of AI chatbots reinforces a broader shift in how responsibility for health risks is allocated (23). By framing users as informed, autonomous decision-makers, such systems promote a model in which individuals are expected to manage their own health. Effectively, this not only relocates the burden of medical decision-making from regulated professionals to lay-users (24), but also shifts responsibility for health away from public services onto individuals (25, 26). While access to health information is an essential part of care, it does not equate to access to health services, and users cannot be expected to understand complex medical information in the same manner as health professionals (27).

Finally, the use of health-focused generative AI may result in a two-tiered system of care. For well-resourced individuals, AI advice can function as a preliminary step before seeking treatment. For individuals who lack adequate access to healthcare, AI systems may instead operate as substitutes for professional services (28). This asymmetry can reinforce health disparities, in particular if AI systems reproduce biases or perform unevenly across demographic groups, as emerging scholarship on conversational agents and health equity also suggests (27, 29). Those already facing barriers to care would be more exposed to misdiagnosis, under-triage, or over-reassurance (30). Because they also have fewer opportunities to verify or override AI outputs through subsequent consultation, errors are less likely to be corrected (27, 31), resulting in a feedback dynamic in which unequal reliance and biased outputs interact to reinforce or deepen inequities in access, safety, and ultimately health outcomes (32). These dynamics present the core challenge of shadow health systems. The concern is not limited to unreliable or inaccurate outputs or advice inconsistent with clinical guidelines (24). Rather, widespread adoption of health-focused generative AI could gradually reroute health information flows through private infrastructures (10, 33, 34). This would give technology providers significant influence over how health information is interpreted, how care is accessed, and how risks are allocated. In doing so, they may reshape healthcare practices, challenge expertise, and deepen inequity without being subject to the same accountability mechanisms that apply to healthcare institutions and professionals (35).

## 4 Gaps in EU digital health regulation

These public health concerns are compounded by a structural legal mismatch. Systems such as ChatGPT Health blur the boundaries between medical and consumer technology, performing health-system functions without clearly falling under healthcare-specific safeguards. At the same time, the initial exclusion of EEA, UK, and Swiss users from early access to ChatGPT Health suggests that providers anticipate regulatory friction at exactly this grey area. Health-focused generative AI chatbots are thus not unregulated. Yet we contend they are governed by legal frameworks that are not suitable to address the public health risks identified above. This section examines how this misalignment arises across different areas of EU law.

### 4.1 Safety and efficacy of health technologies

The first issue concerns safety and performance of health-focused generative AI chatbots. Under EU law, medical software is generally regulated under the Medical Devices Regulation (MDR). The MDR applies when the manufacturer *intends* software to have a medical purpose, for instance diagnosis or treatment. It explicitly excludes wellness and fitness apps from its scope (36). The EU Court of Justice confirmed that a product falls within medical devices law only when it is specifically intended by the manufacturer to be used for a medical purpose (37). As a result, manufacturers can, through disclaimers and product descriptions, avoid application of the MDR by arguing that their product was not intended to have a medical function. This results in a regulatory asymmetry where tools performing similar functions may face different obligations, depending on the declared intended purpose (35).

When an AI chatbot does qualify as a medical device, the MDR imposes various quality and safety rules, including clinical evaluation, quality and risk management systems, documentation, conformity assessments and post-market surveillance (38), safeguards developed precisely to address risks related to efficacy, quality and safety (39). This qualification is also decisive for the scope of application of the Artificial Intelligence Act (AI Act), which applies more stringent rules for AI systems deemed to pose higher risks. In general, medical device AI systems covered by the MDR are considered high-risk AI systems (38). This high-risk classification adds additional, and considerable, obligations related to risk management, bias mitigation, data governance, and documentation, specific to AI risks (37). Thus, if a health-focused generative AI system escapes the MDR qualification, it also avoids the rules for high-risk systems under the AI Act. This leaves only the AI Act’s general-purpose AI regime applicable, which does not require a demonstration of clinical validity or real-world safety and efficacy (40). It does pose the obligation to inform users they are interacting with an AI system and not a human (41, 42). Moreover, the prohibited risk regime will still be applicable, which prohibits purposefully manipulative and exploitative AI systems (43).

This legal gap directly contributes to the public health risks outlined in the previous section. When health-focused AI chatbots operate outside medical device regulation, the responsibility for assessing the reliability of their advice effectively shifts to users (44, 45). Transparency obligations and disclaimers offer limited protection, as repeated, personalized interaction with AI can create a sense of credibility that exceeds lay-users’ ability to evaluate the quality of information (15). At the system level, such allocation of responsibility can lead to delays in seeking care or increased workload for healthcare personnel (29). Moreover, avoiding high-risk classification under the AI Act also circumvents the (limited) anti-discrimination rules under the AI Act (46), which may reinforce existing health disparities.

To close this gap, the European Commission could clarify that software processing clinical-grade health data and producing personalized health guidance is presumed to have a medical purpose under the MDR, unless explicitly demonstrated this is not the case. One pathway is adding these systems to Annex XVI of the MDR, which lists products without an intended medical purpose, to which the MDR nevertheless applies (47). Another pathway is to adopt a delegated act to add certain uses of health-focused AI to Annex III of the AI Act, bringing them under the high-risk regime without MDR classification (48, 49).

Yet, even when these rules do apply, they do little to address the distributive consequences of medical AI. These EU safety frameworks focus on technical performance, but largely ignore how AI adoption may differentially affect populations. To mitigate this, sector-specific safety regimes should require evaluations of medical AI systems that explicitly assess equity effects, helping ensure that AI deployment does not contribute to entrenching two-tiered healthcare systems. This remains challenging in the context of continuously evolving AI systems, as increasingly recognised in the health technology assessment literature (45).

### 4.2 Health claims without accountability

The second gap concerns legal safeguards governing the accuracy and reliability of health information produced by health-focused generative AI chatbots. When medical devices laws do not apply, systems like ChatGPT Health primarily fall under general consumer protection frameworks that prohibit misleading, dangerous, or manipulative products or claims. However, these regimes are not designed to address the public health risks that arise when AI systems begin to function as sources of health advice.

For example, under the General Product Safety Regulation (GPSR), the safety of digitally connected products must be assessed against relevant standards, certification schemes, or good practices, including risks to health. However, these standards are not (yet) developed for AI chatbots, especially in the health domain (50). EU consumer law may also address misleading medical-like claims, omissions, or manipulative interface design, but this regime primarily protects consumers’ economic interests rather than public health considerations. The Digital Services Act (DSA) provides another potential avenue for protection by regulating systemic misinformation risks, including to health, by imposing risk mitigation obligations on very large online platforms. However, ChatGPT currently falls outside of the scope of the DSA’s most stringent obligations (26).

The public health concern therefore lies not merely in the possibility that users may receive incorrect information, but in the structural positioning of these systems as stand-ins for regulated care. By presenting personalized outputs that resemble clinical advice, AI chatbots can acquire an aura of medical authority without being subject to healthcare laws or standards of clinical validation. Consumer law offers some tools to mitigate these risks, including warning obligations and restrictions on misleading claims, but remains limited in addressing the broader transition of epistemic authority in healthcare, as it does not – for example – require providers to systematically correct medically incorrect assumptions or demonstrate ex ante clinical validation (45). Again, this shifts the responsibility for assessing the reliability of their advice to users.

To address this gap, harmonized standards under the GPSR should be developed specifically for health-focused generative AI chatbots (51), requiring demonstrable performance against clinically relevant quality benchmarks. These standards should focus on the functional role of systems providing personalized health-related guidance rather than relying exclusively on formal product labels or declared intended purpose. In parallel, the planned modification of the consumer law regime (including the Digital Fairness Act) should clarify that presenting personalized health outputs in a manner that creates a reasonable impression of clinical reliability without adequate validation constitutes a misleading practice (52).

### 4.3 The protection of health data

Lastly, deficiencies in EU’s data governance are particularly relevant because ChatGPT Health’s central functionality lies in linking health data across contexts. As discussed in Section 3, these AI systems operate not only through advice but also through the data infrastructures that collect, aggregate, and repurpose health data outside regulated care settings. This raises important questions about health data protection. Transferring data from clinical contexts into privately-run AI systems may create privacy risks because the norms and expectations governing data flows in these contexts differ substantially (53). In regulated healthcare settings, providers are bound by ethical and professional duties such as medical confidentiality, therapeutic-purpose limitations, and public interest obligations, that constrain how health data may be used.

In contrast, the processing of health data within generative AI chatbots is primarily governed by contractual terms and consent requirements under the General Data Protection Regulation (GDPR). This framework may, depending on the design of the service, require the providers to ensure data protection by design and by default in accordance with Article 25 GDPR. However, although the GDPR provides enhanced protection for health data, including by requiring a data protection impact assessment for large scale processing of sensitive data under Article 35, reliance on (informed) consent as the main legal basis for integrating health data in the context of a shadow health system seems particularly problematic. As previously discussed, public health implications extend beyond the individual user, yet individuals are made responsible to decide on collective dimensions of data processing. Considering the inability of platform’s privacy policies to meaningfully address relevant information asymmetries (54), expecting users to make these informed decisions is misguided. Article 22 GDPR may also become relevant where AI chatbots are used in ways that produce automated assessments with significant effects, although it is debated whether this safeguard also applies to medical settings (55).

Further, because ChatGPT Health processes highly sensitive personal data, serious risks may arise regarding data breaches and further commercial use of such data (56). Although OpenAI states data security is a priority and claims that health data processed through ChatGPT Health will not be used to further train its AI models, the trustworthiness (and enforceability) of such claims remains uncertain. Furthermore, even if these systems comply formally with existing data protection rules, this does not resolve the public health issues identified above. By mediating healthcare practices and aggregating health data within privately governed infrastructures, these systems may redirect both informational and economic value toward private providers. As a result, any benefits generated from processing health data may be channeled towards companies like OpenAI, rather than returning to the public healthcare systems from which much of this data originated (33).

To remedy these regulatory limitations, EU regulators should clarify that large-scale aggregation of health data and medical records within generative AI advice systems triggers safeguards beyond GDPR compliance. In particular, stricter limits should apply to the secondary commercial use of such data, including AI training, model improvement, profiling, targeted advertising, or commercial partnerships with third parties, alongside reinforced data minimization requirements considering the longitudinal integration of health data across contexts. These safeguards should aim to reflect institutional data safeguards in formal healthcare settings. Closer cooperation between data protection and health authorities should also become the norm, reflecting the hybrid nature of these systems as both digital services and quasi-health infrastructures.

Moreover, the data protection framework should clearly establish that health data collected through AI chatbots for the purpose of providing health advice cannot be repurposed for AI training and development unless users are presented with a separate, explicit, and granular choice that enables genuinely informed consent. Such consent should also remain independent from access to additional services, discounts, or other incentives that could undermine voluntariness (57). Such safeguards are particularly important in light of ongoing policy initiatives that may broaden the lawful bases for data reuse in the name of innovation and regulatory simplification (58). Finally, given that the large-scale processing of health data may generate significant economic value for private providers, policymakers should also consider benefit-sharing mechanisms (including targeted taxation or data value return schemes) to ensure that value derived from health data ultimately flows back to the public systems from which much of this data originates (59).

## 5 Conclusion and the road ahead

This paper has argued that health-focused generative AI chatbots such as ChatGPT Health give rise to what we describe as shadow health systems: privately governed infrastructures that increasingly mediate – and sometimes substitute – engagement with regulated healthcare, without being embedded in corresponding legal and institutional safeguards. In this Policy Brief, we provided a conceptual reflection on their possible public health implications and regulatory positioning. We suggest that by shaping symptom interpretation, triage decisions, care-seeking behavior, and access pathways, shadow health systems can influence healthcare utilization, redistribute responsibility to individuals, and reinforce structural inequities. As these technologies evolve, empirically assessing their real-world effects will be paramount. We also found that EU law is currently unequipped to adequately address these systems’ functions and structural effects. As a result, tools that perform health-system roles can avoid health technology-specific obligations, while remaining governed mainly by consumer and data protection frameworks that are not designed to ensure clinical reliability, equity monitoring, or institutional accountability. If these gaps persist, shadow health systems may become embedded in everyday practice while their power to affect public health remains unchecked.

The regulatory objective should therefore not be to prohibit consumer-facing health AI, but to align legal obligations with functional impact. Where AI systems operate as personalized health advisors at scale, they should be subject to proportionate duties of safety validation, transparency, equity monitoring, and accountability, irrespective of disclaimers or formal product labels. This requires clarifying medical device presumptions, extending high-risk designations where appropriate, developing harmonized safety standards for conversational health AI, and strengthening the limits on health data reuse. More fundamentally, it requires recognizing that health-focused generative AI chatbots are a matter of public health governance, not merely product regulation (33), necessitating the activities of their providers are transparent, justifiable to the public, and contestable (34). Implementing these measures will require coordination between European and national regulators, including medical device authorities, data protection authorities, and consumer protection bodies, as well as careful consideration of enforcement capacity and regulatory overlaps. Ensuring that these emerging infrastructures are aligned with principles of universality, equity, and solidarity is thus essential to preserving a “distinctly European” approach to healthcare (60).
